# Can single progesterone concentration predict miscarriage in early pregnant women with threatened miscarriage: a systematic review and meta-analysis

**DOI:** 10.1186/s12884-024-06303-7

**Published:** 2024-02-13

**Authors:** Yi Gong, Tong Jiang, Yang Sun, Guo-Lin Wu, Bu-Wei Han, Ying Shi, Shan Guan, Jian Li

**Affiliations:** 1 Beilun District People’s Hospital, Beilun Branch of the First Affiliated Hospital of Zhejiang University, Ningbo, China; 2 Guizhou Medical University, Guiyang, China; 3 College of Pharmacy, Harbin University of Commerce, Harbin, China; 4 Department of Obstetrics & Gynecology, Affiliated Hospital of Guizhou Medical University, Guiyang, 150000 China

**Keywords:** Threatened miscarriage, Serum progesterone, Prediction, Miscarriage, Meta-analysis

## Abstract

**Background:**

About 25% of pregnant women experience bleeding in the early stage, and half of them eventually progress to pregnancy loss. Progesterone serves as a useful biomarker to predict miscarriage in threatened miscarriage, yet its performance is still debated.

**Aim:**

To evaluate the performance of single serum progesterone predicting miscarriage in early pregnant patients with threatened miscarriage.

**Method:**

The online database was searched to yield the literature using the terms of ‘Abortion’, ‘Miscarriage’, and ‘serum Progesterone’, including PubMed, Scopus, Embase, Cochrane library, and China national knowledge infrastructure. Receiver operating characteristic (ROC) curve, likelihood ratio (LLR) and diagnostic odds ratio (DOR) and 95% confidence interval (CI) were computed. Publication bias was assessed by the deeks funnel plot asymmetry test. Subgroup analyses were conducted according to the progesterone level (< 12 ng/mL), recruited location and region, progesterone measurement method, exogenous progesterone supplement and follow up.

**Results:**

In total, 12 studies were eligible to be included in this study, with sample sizes ranging from 76 to 1087. The included patients’ gestational age was between 4 and 12 weeks. No significant publication bias was detected from all included studies. The threshold of progesterone reported ranged from 8 to 30 ng/ml. The synthesized area under the ROC curve (0.85, 95% CI 0.81 to 0.88), positive LLR (6.2, 4.0 to 9.7) and DOR (18, 12 to 27) of single progesterone measurement distinguishing miscarriage were relatively good in early pregnant patients with threatened miscarriage. When the threshold of < 12 ng/mL was adapted, the progesterone provided a higher area under the ROC curve (0.90 vs. 0.78), positive LLR (8.3 vs. 3.8) and DOR (22 vs.12) than its counterpart (12 to 30 ng/mL).

**Conclusion:**

Single progesterone measurement can act as a biomarker of miscarriage in early pregnant patients with threatened miscarriage, and it has a better performance when the concentration is <12 ng/mL.

**Trial registration:**

PROSPERO (CRD42021255382).

**Supplementary Information:**

The online version contains supplementary material available at 10.1186/s12884-024-06303-7.

## Introduction

About 25% of pregnant women experience bleeding in the early stage, and half of them eventually progress to pregnancy loss. Currently, neither effective treatment nor prevention strategy is available to manage this condition [1–3]. Despite some medicines are frequently prescribed in clinical practice including progesterone [4], the latter seems only to benefit early pregnant women with a history of one or more previous miscarriages and vaginal bleeding [5].

The vaginal bleeding during the early pregnancy stage is proven to be strongly associated with miscarriage, which is indeed attributed to luteal insufficiency, leading to low progesterone concentration [1–3]. Progesterone, a critical hormone during pregnancy, reflects the luteal function until 7 weeks of gestation, and thus serves as a biomarker to predict the pregnancy outcome in threatened miscarriage [6, 7]. However, the concentration of progesterone varies largely among individuals and across gestational age [8]. Previously, two meta-analyses indicated that both less than 10 ng/mL and 6.3 ng/mL could predict a non-viable pregnancy in early pregnant women including threatened miscarriage [9, 10]. Nevertheless, it is still unclear whether a single progesterone measurement can predict miscarriage in early pregnant women with threatened miscarriage, because most of studies recruited mixed patients including biochemical pregnancy, ectopic pregnancy and threatened miscarriage.

Therefore, we conduct a diagnostic meta-analysis to evaluate the performance of single progesterone measurement predicting miscarriage in early pregnant women with threatened miscarriage.

## Methods

### Protocol and registration

This meta-analysis was registered on PROSPERO (CRD42021255382) and prepared according to systematic reviews and meta-analyses (PRISMA) guidelines [11].

### Literature search

We conduct a comprehensive literature search from Jan 1, 2000 to March 31, 2023, without limitation in language. We searched PubMed, Embase, Scopus, Cochrane Library and China national knowledge infrastructure (CNKI), with the terms of ‘Miscarriage’, Abortion’ and ‘serum Progesterone’. The search strategy used on PubMed-Medline was listed in (Supplement Table 1) in detail. Other databases were tailored when it was necessary, according to the strategy used on PubMed-Medline.

**Table 1:** Characteristics of individual studies included in the meta-analysis

Authors (year)	Country	Sample (N)	Prospective	Recruit patients			Detection of progesterone	Cut-off ng/mL	Supplement	Data extraction					Outcome assessment (weeks)	
				Location	Age (years)	Gestation (weeks)				Tp	Fp	Fn	Tn		Time	Method
Kadam [21] (2019)	India	150	Yes	Outpatient	UK	< 12	Enzyme	11	UK	41	17	4	88		< 13	Ultrasound
Kant [20] (2015)	India	100	Yes	Outpatient	20–39	< 12	Enzyme	24	UK	16	3	5	76		20	Follow up
Ku [26] (2015)	Singapore	119	Yes	Emergency	> 19	6–10	CL	11	UK	23	11	7	78		< 17	Follow up
LeK [24] (2017)	Singapore	345	Yes	Emergency	> 21	6–10	CL	11	UK	46	22	24	253		16	Follow up
Jia [23] (2018)	Singapore	118	Yes	Emergency	21–45	6–10	CL	11	Yes	10	8	5	95		< 16	Follow up
Tan [25] (2020)	Singapore	1087	Yes	Emergency	UK	5–12	CL	11	Yes	170	70	81	766		16	Follow up
Duan [16] (2011)	China	175	No	Outpatient	UK	4–5	Enzyme	16	Yes	51	32	16	76		Live birth	Follow up
Wei [18] (2022)	China	141	UK	Hospitalized	22–43	5–12	CL	22	Yes	16	10	10	105		16	Follow up
Zheng [19] (2021)	China	173	UK	Outpatient	> 20	5–6	CL	10	Yes	38	21	14	100		12	Follow up
Li [17] (2019)	China	100	No	Outpatient	22–35	5–7	UK	10	Yes	18	10	8	64		12	Follow up
Shen [15] (2013)	China	76	UK	Outpatient	UK	< 12	CL	30	Yes	38	7	6	25		Live birth	Follow up
McLindon [22] (2023)	Australia	133	Yes	Outpatient	> 18	< 10	UK	8	Yes	8	2	15	108		Live birth	Follow up
		128							No	4	1	17	106			
		133						16	Yes	17	42	6	68			
		128							No	14	41	7	66			

TP = True Positive, FP = False Positive, TN = True Negative, FN = False Negative

Enzyme = Enzyme Immunoassay. CL: Chemiluminescence

UK = unknown

### Study selection

Inclusion criteria were patients had an ultrasound-confirmed intrauterine pregnancy with gestational age < 12weeks, presented with vaginal bleeding with or without abdominal pain, measured progesterone concentration, reported threshold of progesterone predicting pregnancy outcomes including miscarriage, designed as observational studies or intervention studies (randomized controlled trials), with sample size over 50. Exclusion criteria were women who planned to terminate the pregnancy or have an inevitable miscarriage, got pregnancy via assisted reproductive technologies, took exogenous progesterone prior study, and without threatened miscarriage.

### Data extraction and quality assessment

Two authors (YG and TJ) independently extracted the data from included studies, including the study country, sample size, study design, gestation age, progesterone assessment method, diagnostic threshold of progesterone, exogenous progesterone used during study, true and false positive, true negative and false negative, follow up.

The quality of included studies was evaluated according to the QUADAS-2 standard for quality assessment of diagnostic accuracy [12], including the risk of prejudice and applicability concerns. The risk of bias is consisted of four aspects, namely, patient selection, index testing, reference standards and flow and timing. Applicability assessment included patient selection, index testing, and reference standards.

### Outcomes assessment

The main outcomes included cumulative area under receiver operating characteristic (ROC) curve, sensitivity, specificity, positive and negative likelihood ratio (LLR), , and diagnostic odds ratio (DOR).

### Statistical analysis

STATA17 software (Stata Corp LLC, College Station, Texas, USA) was employed to conduct the statistical analysis when there were 4 or above available dataset, using the ‘midas’ command [13, 14], otherwise, the analysis was conducted by the Metadisc1.4 software. The area under the ROC curve, LLR and DOR and the 95% confidence interval (CI) were calculated. Publication bias was assessed by the deeks funnel plot asymmetry test. Model fits were assessed by quantile plot of residual based goodness-of fit and chi-squared probability plot of squared mahalanobis distances. Subgroup analysis was conducted, stratifying by the threshold of progesterone, recruited location and region, progesterone measurement method, exogenous progesterone supplement and follow up.

## Results

### Characteristics of included studies

**Fig. 1:**
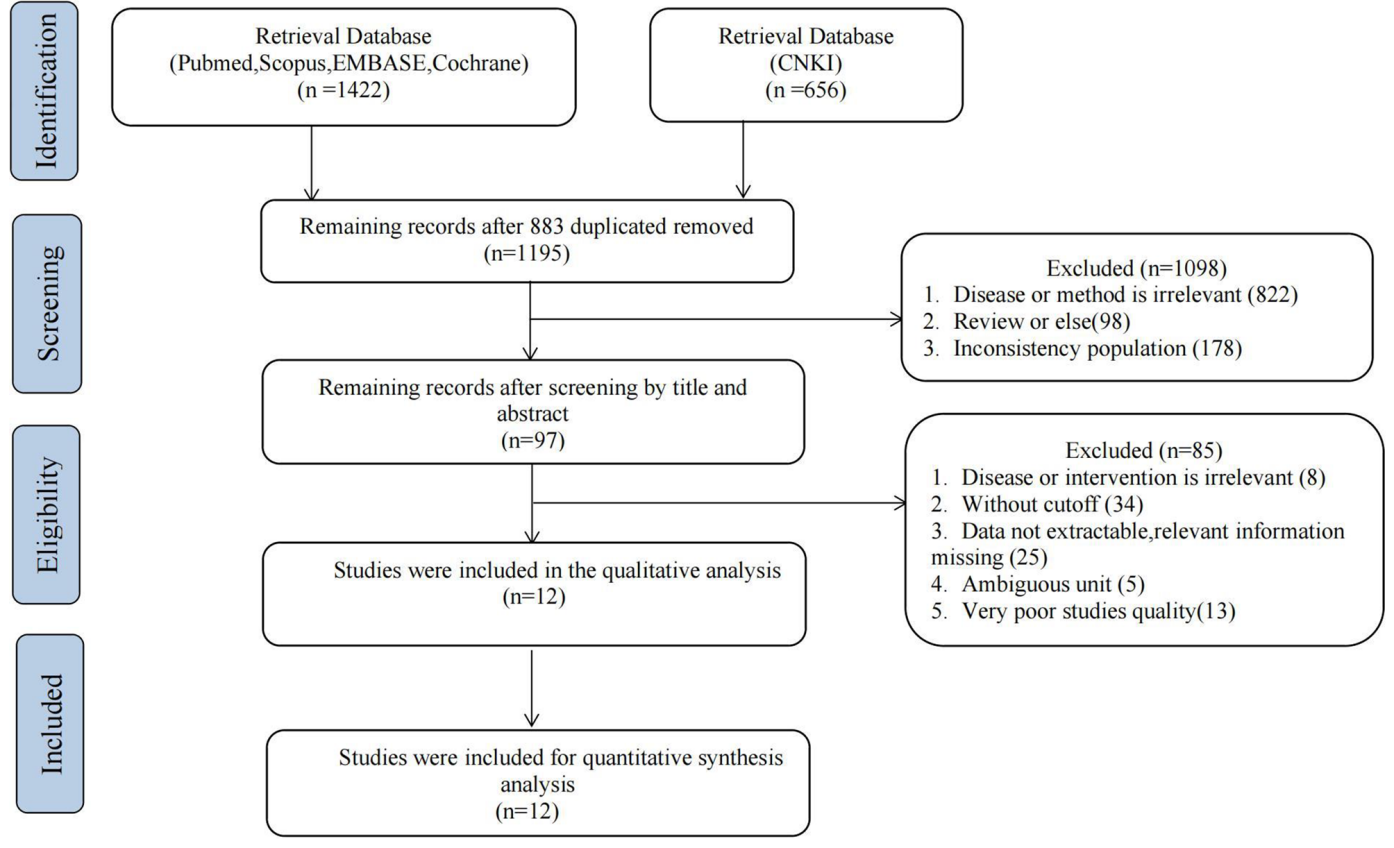
PRISMA flowchart

Overall, 12 studies were included in this study (Fig. 1), and 15 datasets were available to pool the results. The included studies were conducted in India, Singapore, China, and Australia, with a sample size ranging from 76 to 1087. The included patients’ gestational age was between 4 and 12 weeks. The threshold of progesterone reported ranged from 8 to 30ng/ml. Other characteristics of included studies were shown in (Table 1) in detail.

### Quality assessment

The methodological quality was assessed by the QUADAS-2 tool (Table 2). One study did not describe exclusion criteria [15]. Two studies reported as retrospective studies without any information in detail [16, 17] and three studies did not report the study design [15, 18, 19]. One study was judged with a high risk of bias and four were unclear. The selection of diagnostic thresholds was not stated prior the study in 5 studies [16, 18–21] and thus were classified as unclear risk for publication bias. , The outcome assessment was conducted by the ultrasound or follow-up of < 20 weeks of gestation in 9 studies, but live birth in 3 studies [15, 16, 22].  Due to migration, not all patients were included to perform the analysis in 3 studies [22–24]. No significant publication bias was detected (Supplement Fig. 1).

**Table 2:** Quality assessment of included studies

Study	Risk of bias				Applicability Concerns		
	Patient Selection	Index Test	Reference Standard	Flow and Timing	Patient Selection	Index Test	Reference Standard
Duan et al. [16]	U	U	U	L	L	L	H
Jia et al. [23]	L	L	L	U	L	L	L
Kadam et al. [21]	L	U	L	L	L	L	L
Kant et al. [20]	L	U	L	L	L	L	L
Ku et al. [26]	L	L	L	L	L	L	L
LeK et al. [24]	L	L	L	U	L	L	L
Li et al. [17]	U	L	L	L	L	L	L
McLindon et al. [22]	L	L	U	U	L	L	H
Shen et al. [15]	H	L	U	L	U	L	H
Tan et al. [25]	L	L	L	L	L	L	L
Wei et al. [18]	U	U	L	L	L	L	L
Zheng et al. [19]	U	U	L	L	L	L	L

H = high risk of bias, L = low risk of bias, U = unclear risk of bias

### Diagnostic performance

**Fig. 2:**
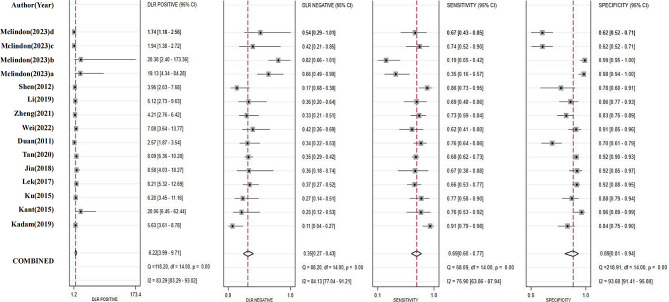
Diagnostic test accuracy for single study

**Fig. 3:**
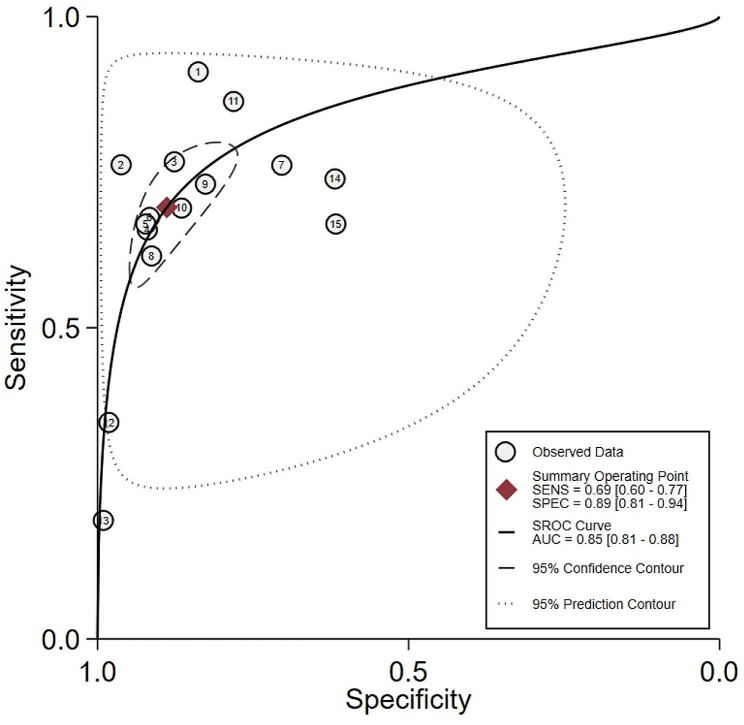
Summary area under the ROC Curve

The sensitivity, specificity, positive LLR and negative LLR of progesterone in each study were summarized (Fig. 2). The pooled sensitivity (0.69, 95% CI 0.60 to 0.77) and specificity (0.89, 95% CI 0.81 to 0.94) of progesterone predicting miscarriage were relative good, providing positive LLR (6.2, 95% CI 4.0 to 9.7) and negative LLR (0.35, 0.27 to 0.43). The synthesized area under the ROC curve (0.85, 95% CI 0.81 to 0.88) had similar performance (Fig. 3).

### Subgroup analysis

The threshold of < 12 ng/mL was reported in 8 studies [17, 19, 21–26], the rest ranging from 12 to 30 ng/mL [15, 16, 18, 20, 22]. When the threshold of < 12 ng/mL was adapted, progesterone yielded a higher ROC (0.90 vs. 0.78), positive LLR (8.3 vs. 3.8) and DOR (22 vs.12) than its counterpart (12 to 30 ng/mL), respectively (Table 3). Other factors including recruited location and region, progesterone measurement method, exogenous progesterone supplement and follow up, did little impacts on the diagnostic performance.

**Table 3:** Summarized results of the meta-analysis

	Dataset(n)	ROC	Sensitivity	Specificity	Positive likelihood ratio	Negative likelihood ratio	Diagnostic odds ratio	Threshold effect (P value)	Heterogeneity (I^2^)
All combined	15	0.85(0.81–0.88)	0.69(0.60–0.77)	0.89(0.81–0.94)	6.2(4.0-9.7)	0.35(0.27–0.43)	18(12–27)	0.85	98%
Subgroup analysis									
Threshold value(ng/mL)									
<12	9	0.90(0.87–0.92)	0.65(0.49–0.77)	0.92(0.87–0.95)	8.3(5.9–11.7)	0.38(0.27–0.55)	22(16–29)	1.00	96%
12–30	6	0.78(0.74–0.81)	0.75(0.68–0.81)	0.80(0.65–0.90)	3.8(2.0-7.1)	0.31(0.24–0.41)	12(5–27)	0.45	86%
Recruit patients									
Outpatient	10	085(0.81–0.88)	0.69(0.55–0.81)	0.88(0.75–0.94)	5.6(2.9–10.6)	0.35(0.24–0.51)	16(9–30)	0.66	98%
Hospitalized or Emergency	5	0.89(0.86–0.92)	0.68(0.63–0.72)	0.91(0.90–0.93)	7.9(6.6–9.5)	0.35(0.31–0.41)	22(17–30)	0.70	100%
Measurement method									
Enzyme*	3	0.90	0.81(0.74–0.88)	0.82(0.77–0.86)	5.7(2.3–13.9)	0.23(0.12–0.44)	29(6-145)	0.67	*NA*
Chemiluminescence	7	0.88(0.85–0.90)	0.72(0.65–0.78)	0.89(0.86–0.92)	6.7(5.1–8.6)	0.32(0.26–0.39)	21(16–28)	1.00	67%
Progesterone supplement									
Yes	9	0.83(0.79–0.86)	0.71(0.62–0.78)	0.87(0.78–0.93)	5.5(3.3-9.0)	0.34(0.28–0.41)	16(11–25)	1.00	97%
No or unclear	6	0.88(0.85–0.91)	0.69(0.48–0.84)	0.91(0.78–0.97)	7.9(3.4–18.2)	0.34(0.20–0.60)	23(10–55)	0.53	96%
Out of China									
No	10	0.86(0.82–0.89)	0.66(0.52–0.78)	0.91(0.82–0.96)	7.6(4.0-14.4)	0.37(0.26–0.52)	21(11–37)	0.69	98%
Yes	5	0.84(0.80–0.87)	0.74(0.65–0.81)	0.82(0.74–0.89)	4.2(2.9–6.1)	0.32(0.24–0.41)	13(8–21)	1.00	76%
Follow up (weeks)									
≤20	9	0.89(0.86–0.92)	0.72(0.66–0.78)	0.90(0.87–0.92)	7.1(5.8–8.6)	0.31(0.25–0.38)	23(18–30)	1.00	76%
>20	6	0.80(0.76–0.83)	0.63(0.40–0.81)	0.87(0.61–0.96)	4.7(1.8–12.0)	0.43(0.28–0.66)	11(5–23)	0.96	98%

*The model fitting was performed with Metadisc software (1.4), because of the limitation of the dataset number

## Discussion

### Main results

This study indicated that single progesterone measurement can act as a biomarker predicting miscarriage in early pregnant women with threatened miscarriage, and the performance was better when the concentration was < 12 ng/mL.

### Strengths and limitations

In this updated meta-analysis, we included 12 studies which focused on investigating the progesterone predicting miscarriage in early pregnant women with threatened miscarriage, providing a more robust evaluation compared with the previous one [27]. In addition, as the incidence of threatened miscarriage varies widely across races, regions and countries, the use of sensitivity and specificity to assess progesterone predicting miscarriage in this condition is inappropriate and inaccuracy [28]. Therefore, together with sensitivity and specificity, the LLR was calculated [29], because the latter had been proposed to be an prefer outcome in diagnostic study [30, 31].

Also, there are several limitations in this study. First, the threshold interval was relatively wide, lowering the accuracy of estimation. Second, high heterogeneity was observed among studies, leading to increased risk of diagnostic bias and unstable evaluation. Last but not least, a meta-regression analysis can not be performed, due to the absent available data and limited studies.

### What ideas does our research provide for clinical applications

Previously, it had been reported that about 50% of women facing threatened miscarriage were affected depressive and anxiety symptomatology [32]. When it comes to the case, they might benefit from an effective treatment or prevention strategy, due to the fear of pregnancy loss, especially for those who had an experience of miscarriage. Unfortunately, there is still absent an effective treatment or prevention strategy. Despite many experimental treatments are frequently prescribed in the real world including exogenous progesterone supplement, the latter had been demonstrated did not improve the clinical outcomes in this subpopulation. As the progesterone concentration < 10 ng/mL can predict a non-viable pregnancy [10], the issue is how is the prognostic value of progesterone concentration in early pregnant women with threatened miscarriage, especially for those who initially seek medical care and conduct the progesterone measurement. Our findings suggested that serum progesterone < 12 ng/mL at a single measurement can also effectively predict miscarriage in early pregnant patients with threatened miscarriage. Therefore, progesterone, a useful prognostic biomarker, would provide help to make a decision in practice and reduce the anxiety secondary to the threatened miscarriage.

What progress does our research provide for future research?

During the first trimester, serum progesterone levels fluctuate over a 24-hour period, the concentration at a single measurement would be influenced by a short-term pattern of pulses, distribution of the hormone and the diet [33]. Moreover, 7 to 8 weeks is considered to be the stage of the luteal-placental shift, progesterone levels may be plateaus or even decrease trend [7], attracting the largest attentions during this period with a highest frequency of vaginal bleeding [34]. Whereas few studies explored the performance of progesterone during the period before 7 weeks of gestation. Thus, studies are warranted to be conducted to further validate the prognostic value of progesterone prior 7 weeks of gestation and clarify the optimal time point of single measurement. In addition, the diagnostic performance of progesterone seemed to be little impacted by the exogenous progesterone supplement even thought at a single measurement without specific time point. This deserves to be validated by further studies, given the fact that exogenous progesterone supplement is still widely prescribed in clinical practice [35].

## Conclusion

Single progesterone can act as a prognostic biomarker in early pregnant women with threatened miscarriage, it has a better performance when its concentration is < 12 ng/mL.

### Electronic supplementary material

Below is the link to the electronic supplementary material.

Supplementary Material 1

Supplementary Material 2

Supplementary Material 3

Supplementary Material 4

## Data Availability

All data generated or analyzed during this study are included in this published article.

## References

[1] Everett C (1997). Incidence and outcome of bleeding before the 20th week of pregnancy: prospective study from general practice. BMJ.

[2] Hendriks E, MacNaughton H, MacKenzie MC (2019). First trimester bleeding: evaluation and management. Am Fam Physician.

[3] Greene MF (2019). Progesterone for threatened abortion. N Engl J Med.

[4] Sotiriadis A, Papatheodorou S, Makrydimas G (2004). Threatened miscarriage: evaluation and management. Br Med J.

[5] Devall AJ, Papadopoulou A, Podesek M, Haas DM, Price MJ, Coomarasamy A (2021). Progestogens for preventing miscarriage: a network meta-analysis. Cochrane Database Syst Rev.

[6] Daya S (2009). Luteal support: progestogens for pregnancy protection. Maturitas.

[7] Schindler A (2004). First trimester endocrinology: consequences for diagnosis and treatment of pregnancy failure. Gynecol Endocrinology: Official J Int Soc Gynecol Endocrinol.

[8] Ku CW, Zhang X, Zhang VR-Y, Allen JC, Tan NS, Østbye T (2021). Gestational age-specific normative values and determinants of serum progesterone through the first trimester of pregnancy. Sci Rep.

[9] Ghaedi B, Ameri S, Abdulkarim K, Thiruganasambandamoorthy V. Prognostic value of single serum progesterone in the evaluation of symptomatic pregnant patients: A systematic review and meta-analysis. Can J Emerg Med., Ghaedi B, Ameri S, Abdulkarim K, Thiruganasambandamoorthy V.) Ottawa Hospital Research Institute, Ottawa, ON, Canada:S23–4.

[10] Verhaegen J, Gallos ID, van Mello NM, Abdel-Aziz M, Takwoingi Y, Harb H (2012). Accuracy of single progesterone test to predict early pregnancy outcome in women with pain or bleeding: meta-analysis of cohort studies. BMJ.

[11] Page MJ, McKenzie JE, Bossuyt PM, Boutron I, Hoffmann TC, Mulrow CD (2021). The PRISMA 2020 statement: an updated guideline for reporting systematic reviews. BMJ.

[12] Whiting PF, Rutjes AWS, Westwood ME, Mallett S, Deeks JJ, Reitsma JB (2011). QUADAS-2: a revised tool for the quality assessment of diagnostic accuracy studies. Ann Intern Med.

[13] Dwamena B. MIDAS: Stata module for meta-analytical integration of diagnostic test accuracy studies. Stat Softw Compon. 2009;14 Feb.

[14] Dwamena BA. Meta-analytical integration of Diagnostic Accuracy studies in Stata. Ben Dwamena. 2007.

[15] Shen Hong-yun (2013). Zhang Wen-bing. Clinical value of combined detection of serum progesterone and β-HCG in early threatened abortion. Chin Gen Pract.

[16] Duan L, Yan D, Zeng W, Yang X, Wei Q (2011). Predictive power progesterone combined with beta human chorionic gonadotropin measurements in the outcome of threatened miscarriage. Arch Gynecol Obstet.

[17] Li Qiong Z, Xiao XU, Hai-Geng LU, Jin-fei Z, Xia C (2019). The predictive value of combined detection of β-HCG doubling rate and progesterone in the treatment effect of threatened abortion in euthyroid pregnant women. Mod Chin Doctors.

[18] Wei Hui LI, Ye-hua P, Je-rong MENG, Wen-xia LUO, Liu-Hua (2022). Levels of anti-trophoblast cell membrane antibody, carbohydrate antigen 12 – 5, estrogen and progesterone in patients with threatened abortion in early pregnancy and their predictive efficacy for fetal protection failure. Med Guangxi.

[19] Zheng Xin-yu, Wei LI, Yan-sa ZHAO (2021). Clinical value of peripheral blood insulin-like growth factor-1, transforming growth factor-β1, inflammatory factors and reproductive hormone levels in predicting the outcome of threatened abortion in early pregnancy. Chin Med Frontier J (Electronic Edition).

[20] Kant RH, Ara S, Lone AI, Gupta S (2015). Evaluation of outcome of pregnancy in threatened abortion by serum progesterone levels. Int J Reprod Contracept Obstet Gynecol.

[21] Kadam VK, Agrawal S, Saxena P, Laul P (2019). Predictive value of single serum progesterone level for viability in threatened miscarriage. J Obstet Gynecol India.

[22] McLindon LA, James G, Beckmann MM, Bertolone J, Mahomed K, Vane M (2023). Progesterone for women with threatened miscarriage (STOP trial): a placebo-controlled randomized clinical trial. Hum Reprod.

[23] Siew JYS, Allen JC, Hui CYY, Ku CW, Malhotra R, Østbye T, et al. The randomised controlled trial of micronised progesterone and dydrogesterone (TRoMaD) for threatened miscarriage. European Journal of Obstetrics and Gynecology and Reproductive Biology. 2018;228 (Siew J.Y.S.; Hui C.Y.Y.; Ku C.W.; Tan T.C., tan.thiam.chye@singhealth.com.sg) Department of Obstetrics and Gynaecology, KK Women?s and Children?s Hospital, Singapore:319–24. 10.1016/j.ejogrb.2018.07.028 30077119

[24] Lek SM, Ku CW, Allen JC, Malhotra R, Tan NS, Østbye T (2017). Validation of serum progesterone < 35nmol/L as a predictor of miscarriage among women with threatened miscarriage. BMC Pregnancy Childbirth.

[25] Tan TC, Ku CW, Kwek LK, Lee KW, Zhang X, Allen JC (2020). Novel approach using serum progesterone as a triage to guide management of patients with threatened miscarriage: a prospective cohort study. Sci Rep.

[26] Ku CW, Allen JC, Malhotra R, Chong HC, Tan NS, Østbye T (2015). How can we better predict the risk of spontaneous miscarriage among women experiencing threatened miscarriage?. Gynecol Endocrinol.

[27] Pillai RN, Konje JC, Tincello DG, Potdar N (2016). Role of serum biomarkers in the prediction of outcome in women with threatened miscarriage: a systematic review and diagnostic accuracy meta-analysis. Hum Reprod Update.

[28] Gallagher EJ (1998). Clinical utility of likelihood ratios. Ann Emerg Med.

[29] Khan KS, Khan SF, Nwosu CR, Arnott N, Chien PF (1999). Misleading authors’ inferences in obstetric diagnostic test literature. Am J Obstet Gynecol.

[30] Eusebi P (2013). Diagnostic accuracy measures. Cerebrovasc Dis.

[31] Grimes DA, Schulz KF (2005). Refining clinical diagnosis with likelihood ratios. Lancet.

[32] Zhu CS, Tan TC, Chen HY, Malhotra R, Allen JC, Østbye T (2018). Threatened miscarriage and depressive and anxiety symptoms among women and partners in early pregnancy. J Affect Disord.

[33] Nakajima ST, McAuliffe T, Gibson M (1990). The 24-hour pattern of the levels of serum progesterone and immunoreactive human chorionic gonadotropin in normal early pregnancy. J Clin Endocrinol Metab.

[34] Hasan R, Baird DD, Herring AH, Olshan AF, Jonsson Funk ML, Hartmann KE (2010). Patterns and predictors of vaginal bleeding in the first trimester of pregnancy. Ann Epidemiol.

[35] Pang Y-Y, Ma C-L (2022). Real-world pharmacological treatment patterns of patients with threatened miscarriage in China from 2014 to 2020: a cross-sectional analysis. J Clin Pharm Ther.

