# Supplementary figures and images for: Can single progesterone concentration predict miscarriage in early pregnant women with threatened miscarriage: a systematic review and meta-analysis
Source: BMC Pregnancy Childbirth. 2024 Feb 13;24:133. doi: 10.1186/s12884-024-06303-7 (PMC10863102; doi:10.1186/s12884-024-06303-7)

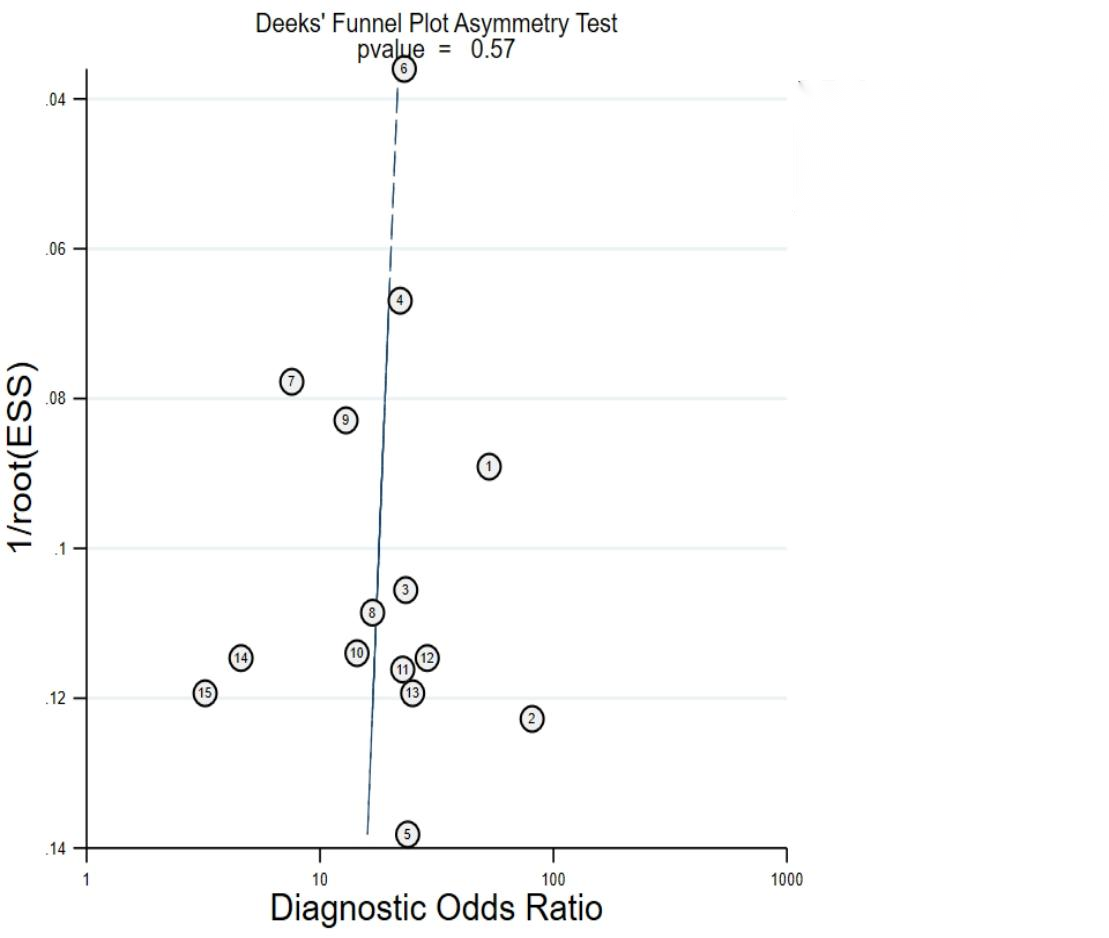


Supplement figure 1. Funnel plot of included studies.

Supplement: Supplementary file 1 — Supplementary Material 1 [file 12884_2024_6303_MOESM1_ESM.docx]
