# Supplementary material for: Can single progesterone concentration predict miscarriage in early pregnant women with threatened miscarriage: a systematic review and meta-analysis
Source: BMC Pregnancy Childbirth. 2024 Feb 13;24:133. doi: 10.1186/s12884-024-06303-7 (PMC10863102; doi:10.1186/s12884-024-06303-7)
Supplement: Supplementary file 2 — Supplementary Material 2 [file 12884_2024_6303_MOESM2_ESM.docx]

| 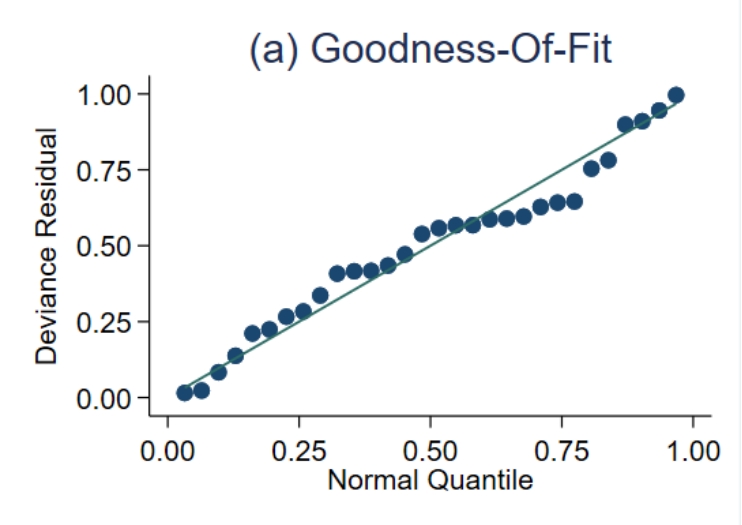  Quantile plot of residual based goodness-of fit  Supplement Figure 2. Model fitting | 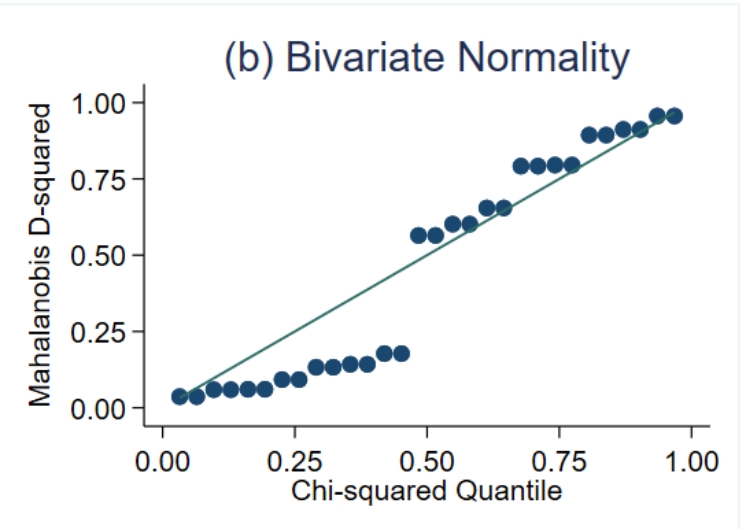  Chi-squared probability plot of squared Mahalanobis distances |
| --- | --- |
