# Supplementary material for: Can single progesterone concentration predict miscarriage in early pregnant women with threatened miscarriage: a systematic review and meta-analysis
Source: BMC Pregnancy Childbirth. 2024 Feb 13;24:133. doi: 10.1186/s12884-024-06303-7 (PMC10863102; doi:10.1186/s12884-024-06303-7)
Supplement: Supplementary file 3 — Supplementary Material 3 [file 12884_2024_6303_MOESM3_ESM.docx]

| Number | Pubmed | Scopus | EMbase | Cochrane | CNKI |
| --- | --- | --- | --- | --- | --- |
| #1 | (((miscarriage  [MeSH Terms]) | KEY(miscarriage) | miscarriage'/exp | Miscarriage/MeSH | serum Progesterone  /subject |
| #2 | (Abortions  [MeSH Terms])) | KEY(abortion) | abortion''/exp | Abortions/MesH | miscarriage/subject |
| #3 | (serum Progesterone  [MeSH Terms])) | KEY(serum AND progesterone)) | serum progesterone'/exp | Serum Progesterone/MeSH |  |
| #4 | (#1 or #2) and #3(374) | (#1 or #2 ) and #3(128) | (#1 or #2) and #3(740) | (#1 or #2) and #3(180) | #1 and #2(656) |

Supplement Table 1. Search strategy
