# Supplementary material for: Can single progesterone concentration predict miscarriage in early pregnant women with threatened miscarriage: a systematic review and meta-analysis
Source: BMC Pregnancy Childbirth. 2024 Feb 13;24:133. doi: 10.1186/s12884-024-06303-7 (PMC10863102; doi:10.1186/s12884-024-06303-7)
Supplement: Supplementary file 4 — Supplementary Material 4 [file 12884_2024_6303_MOESM4_ESM.docx]

Supplement Table 2. The “midas” command was used in the statistics, as follows

|  | Table or figure | command |
| --- | --- | --- |
| 1 | Summarized results( table 3) | midas tp fp fn tn, res(sum) |
| 2 | Diagnostic test accuracy ( figure2) | midas tp fp fn tn, id(author) table(dss) texts(0.60) bfor(dss) ford fors ms(0.75) |
|  |  | midas tp fp fn tn, id(author) table(dlr) texts(0.7) ford fors bfor(dss) |
| 3 | Summary ROC ( figure3) | midas tp fp fn tn, plot sroc(both) |
| 4 | Funnel (supplement figure1) | midas tp fp fn tn, pubbias |
| 5 | Model fitting (supplement figure2) | midas tp fp fn tn, modchk(all) |
